# Sensorimotor Experience Influences Recovery of Forelimb Abilities but Not Tissue Loss after Focal Cortical Compression in Adult Rats

**DOI:** 10.1371/journal.pone.0016726

**Published:** 2011-02-16

**Authors:** Marina Martinez, Jean-Michel Brezun, Christian Xerri

**Affiliations:** CNRS UMR 6149, Integrative and Adaptive Neurosciences, Pôle 3 C, IFR 131, University of Provence, Marseilles, France; University of Muenster, Germany

## Abstract

Sensorimotor activity has been shown to play a key role in functional outcome after extensive brain damage. This study was aimed at assessing the influence of sensorimotor experience through subject-environment interactions on the time course of both lesion and gliosis volumes as well as on the recovery of forelimb sensorimotor abilities following focal cortical injury. The lesion consisted of a cortical compression targeting the forepaw representational area within the primary somatosensory cortex of adult rats. After the cortical lesion, rats were randomly subjected to various postlesion conditions: unilateral C5–C6 dorsal root transection depriving the contralateral cortex from forepaw somatosensory inputs, standard housing or an enriched environment promoting sensorimotor experience and social interactions. Behavioral tests were used to assess forelimb placement during locomotion, forelimb-use asymmetry, and forepaw tactile sensitivity. For each group, the time course of tissue loss was described and the gliosis volume over the first postoperative month was evaluated using an unbiased stereological method. Consistent with previous studies, recovery of behavioral abilities was found to depend on post-injury experience. Indeed, increased sensorimotor activity initiated early in an enriched environment induced a rapid and more complete behavioral recovery compared with standard housing. In contrast, severe deprivation of peripheral sensory inputs led to a delayed and only partial sensorimotor recovery. The dorsal rhizotomy was found to increase the perilesional gliosis in comparison to standard or enriched environments. These findings provide further evidence that early sensory experience has a beneficial influence on the onset and time course of functional recovery after focal brain injury.

## Introduction

Numerous studies have shown that sensorimotor activity influences functional recovery following extensive brain injury (for a review, see [1]). In rats, exposure to an enriched environment (EE) has a beneficial effect on functional outcome [2], [3], whereas the effects of intensive physical activity such as wheel running [2], [3], [4] or forced sensorimotor exercise [5], [6], [7] on behavioral recovery depends on the lesion type, localization, size and/or on onset and duration of sensorimotor exercise. EE, which provides animals with both social interactions and sensorimotor experience, has consistently been shown to improve functional recovery following various methods of inducing injuries to the cerebral cortex, such as a photochemical lesion [8], lateral fluid percussion [9], or damage to both cortical and subcortical structures through middle cerebral artery occlusion [2], [3], [10]. However, the effects of EE on the infarct size and gliosis extent remain poorly understood. Some studies have reported no significant effect of EE on infarct size [2], [9], [10] and gliosis [4], [8], whereas others have demonstrated that EE can lead to increased astrocyte proliferation [11]. It is noteworthy that none of those studies investigated the postoperative time course of the morphological events.

In contrast to EE, reducing sensorimotor experience by transitory immobilization of the affected limb or housing in an impoverished environment was not found to impact significantly the functional deficits or tissue loss in rats with cortical damage [5], [12]. However, studies designed to examine how sensory input influences the recovery from sensorimotor functions following brain injury would benefit from experimental protocols contrasting the effects of naturalistic EE with those of a standard housing environment (SE). Further insight should be gained by assessing the consequences of selective and irreversible deprivation of the cortical area targeted by the lesion. From this perspective, compression of the sensorimotor [13] or somatosensory [14] cortices has been shown to allow for highly reproducible effects in terms of morphological alterations [15] and/or functional impairments. However, those studies were not primarily aimed at investigating the behavioral consequences of such cortical damages, but were mainly focused on the validation of the lesion method.

The purpose of the present study was to evaluate the influence of postoperative sensory experience on the time course of both behavioral recovery and morphological changes following a cortical compression restricted to the forepaw representational area within the primary somatosensory cortex (S1-FL). We assessed the lesion size and associated gliosis as well as the recovery of forelimb sensorimotor abilities in rats exposed to SE, EE promoting somatosensory experience or subjected to dorsal root section selectively depriving the forelimb somatosensory cortex. Another advantage of using dorsal rhizotomy resides in the opportunity to assess unambiguously the contribution of somatosensory inputs to recovery of fine sensorimotor adjustments following cortical damage.

## Materials and Methods

All experiments were carried out in accordance with the National Institute of Health Guide for Care and Use of Laboratory Animals (NIH Publication n° 80–23) revised 1996 for the UK Animals (Scientific Procedures) Act 1986 and associated guidelines, or the Policy on Ethics approved by the Society for Neuroscience in November 1989, and amended in November 1993. No institutional review board or ethics committee are requested to approve animal research in France. The people performing the experiments on the animals are licensed (JMB:A13055-25,13.271; CX:A13055-25,13.86).

### Animals and experimental protocols

**Figure 1:**
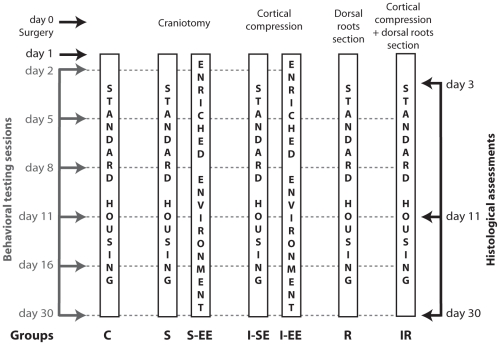
Experimental design. Day 0 refers to the day of surgery. Behavioral evaluations were performed from the 2nd or 5th to the 30th postlesion day. On the 3rd, 11th or 30th postlesion day, rats were euthanized and brains were prepared for further histological analysis.

Three-month-old Long Evans male rats (Janvier Laboratories, France) (n = 80) weighing 300 to 350 g were assigned to seven groups (Fig. 1). The rats were housed on a 12 h light-dark cycle and had access to food and water ad libitum.

- Three groups of rats were housed in standard environment (SE), *i.e.* 3 animals per cage experiencing a moderate level of sensorimotor activity: control rats (C; control), sham rats (S; parietal craniotomy) and injured rats (I-SE; cortical injury).
- Two groups of rats were exposed to an enriched environment (EE) promoting tactile experience as early as 24 hours after surgery and for one month: the injured (I-EE; cortical injury) and sham (S-EE; parietal craniotomy) rats. The EE consisted of large cages (120 cm wide, 120 cm deep, 100 cm high) containing objects of different shapes, sizes, and textures to promote tactile experience [16]. Each day, the EE cages were furnished with a new set of objects to stimulate the exploratory behaviour of the rats. Daily observation of the EE rats indicated that the animals were very active in terms of touching, palpating, and manipulating the various objects composing their physical environment. Tactile stimulation was reinforced with mobile objects and running wheels covered with textures of various roughnesses. Exploratory behavior was found to alternate with grooming and mutual cleaning.
- Two groups of rats were subjected to a section of both C5 and C6 dorsal roots (R; dorsal rhizotomy only; IR; cortical injury and dorsal rhiztomy) and were maintained in standard housing conditions.

Each of these groups included 5 rats which were studied for one month. These rats were used for both behavioral assessments and histological analyses. In addition, 45 rats from the S, I-SE, I-EE, IR and R groups (5 rats per group) were only used for histological analyses and were euthanized 3 and 11 days after surgery, except for the I-EE rats that were not used on the first histological time point, as the placement in EE began 24 hours after the cortical lesion (Fig. 1).

### Surgery

Anesthesia was induced by an intramuscular injection of ketamine (25 mg/kg, Ketalar, Virbac, France) and medetomidine (0.25 mg/kg, Domitor®, Orion Pharma, Finland). An analgesic substance, olfenamic acid (10 mg/kg, s.c.,Tolfédine® Vetoquinol France), was injected 20 minutes before surgery. The core temperature was continuously monitored by a rectal thermistor probe and maintained constant (38±0.1°C) with a feedback-controlled homeothermic blanket system (Harvard Apparatus Ltd., Kent, UK), as variation in temperature has been found to modify the severity of tissue damage following cortical compression [13].

- *Cortical compression:* the surgical procedures were adapted from Kundrotiené et al. [13]. After exposure of the skull by a medial incision of the scalp and the retraction of attached muscles, a parietal craniotomy was performed, centered on the forelimb representation area of the somatosensory cortex. The dura remained intact during all the procedure and was continuously bathed in physiological saline. A curvilinear piston (3×4 mm) was applied over the left somatosensory cortex in the area devoted to the contralateral forepaw representation (S1-FL) (ant.:+1.5; lat.: +4.5; incisor bar: -3.3) with an angle of 20° relative to the vertical axis. These coordinates were been previously determined on the basis of a series of electrophysiological mapping studies [16]. The piston was placed on the dura and slowly moved down along a 3.0 mm distance at the rate of 1 mm/min. The compression was maintained constant for 30 minutes. Then, the piston was removed at the speed of 1 mm/min. Such a compression has been shown to induce a reproducible damage of the cortical tissue, while sparing deeper structures [13]. The state of the brain surface was examined immediately after the compression. Animals exhibiting oedema and/or bleeding were removed from the study. Then, the bone flap was put back and the skin sutured. The S and S-EE rats underwent the surgical procedure described above, but the cortex was not damaged.
- *Dorsal rhizotomy:* after a medial incision of the skin along the dorsal part of the neck, the skin and superficial muscles were retracted. The paravertebral muscles inserted on the dorsal part of the C5 vertebra were dissected and a bilateral laminectomy exposed the dorsal surface of the spinal cord. The dura was incised and droplets of Lidocaine (Xylovet®, CEVA, France) were applied on the spinal cord to decrease spinal reflexes. The C5–C6 dorsal roots were then cut using microscissors under microscope visualization and a 500 µm piece of root was removed to prevent any axonal regrowth. After the surgery, a local antibiotic was applied on the exposed part of the spinal cord (Flumiquil® 3%, CEVA, France), the bone flap was replaced and secured with dental cement. The muscles and skin were sutured using absorbable and non absorbable materials, respectively. In order to evaluate the effects of a C5–C6 dorsal root section on the functional activity of the contralateral somatosensory cortex, electrophysiological multiunit recordings were performed in the somatosensory forepaw representation area of 4 rats, before and 1–2 hours after the lesion. As expected, a C5–C6 dorsal rhizotomy induced a complete abolishment of somatosensory-evoked responses within the forepaw area, while spontaneous discharges still persisted, indicating that the distal part of the forelimb was completely deafferented.

### Histology and immunochemistry

To assess the influence of sensory inputs on the time course of cortical tissue loss, three survival periods were used in the rats subjected to the cortical compression (*i.e.* I-SE, I-EE and IR rats) as well as in the R and S rats. After a 3, 11 or 30 day period, the animals were given a lethal dose of pentobarbital sodium (150 mg/kg, i.p., CEVA) and transcardially perfused with cold saline followed by 500 ml of freshly prepared cold 4% paraformaldehyde (PFA) in 0.1 M phosphate buffer (PB, pH 7.4). Then, the brains were carefully dissected out and the tissue was post-fixed for 12 h at 4°C in 4% PFA. The blocks were cryoprotected by successive transfers into increasing concentrations (10%, 20% and 30%) of sucrose solution in 0.1 M phosphate buffer saline (PBS) for 72 h at 4°C. For histological examination, the brains were frozen using carbon dioxide and 40-µm-thick coronal sections centered on the cortical lesions were performed using a cryostat. Every 6th section was mounted on slides, then stained with cresyl violet. Adjacent sections were processed for an immunodetection of the glial fibrillary acidic protein (GFAP). Free-floating sections were pre-incubated for 30 minutes in lysine (18.3 mg/ml, Sigma, France) and H_2_O_2_ (1%) and then for 30 minutes in blocking buffer (10% goat or 5% swine normal serum, Dako, France) containing 0.3% Triton X100. The incubation with the primary antibody against GFAP (Dako, france) was performed for 24 hr at 4°C, in presence of 0.3% Triton X100 and 1% normal serum. Sections were then incubated with biotinylated secondary antibodies (Abcys, France) with 1% normal serum, followed by an Avidine-Biotin-Complex staining (Vector) for 2 hour at room temperature. Between every stage, rinses were performed with PBS containing normal serum (1%, 3×8 min). Peroxidase activity was revealed by incubating sections with 0.025% 3.3′-diaminobenzidine (DAB, Sigma, France) and 0.01% H_2_O_2_ in 0.5 M tris buffer saline (TBS) pH 7.6. The sections were processed in parallel and the incubation times with chromogene were carefully controlled. After several rinses, sections were mounted on gelatin-coated slides, dehydrated, and cover-slipped in a mounting medium. Omission of the primary antibodies resulted in lack of specific immunolabeling. Photomicrography and light microscopic examinations were performed with a Nikon (Paris, France) microscope. Sections were digitally imaged and analyzed for an unbiased stereological estimation of tissue loss (cresyl violet staining) and gliosis (GFAP immunoreactivity, GFAP-Ir) volumes by using the Cavalieri method (Mercator software, Explora Nova, France). On each section quantified, the gliosis area was assessed by measuring the perilesional zone in which an enhancement of GFAP-Ir was observed. For each volume estimate, the coefficient of error was also determined.

### Behavioral testing

The animals were handled each day over a 2-week period to acclimate them to the behavioral devices and thereby ensure measurement reliability over subsequent testing sessions. Behavioral testing was then conducted over a 4-week period post-injury (2, 5, 8, 11, 16 and 30 days) in all groups surviving 30 days, except for the IR and R groups that were tested only from the 5th to the 30th postoperative day (Fig. 1).

- *Horizontal ladder beam walking test:* forelimb deficits were assessed using the ladder rung walking test apparatus [17], [18]. The animals were trained to walk on the ladder (200 cm long with rungs of 3 mm diameter) from a neutral cage to their home cage while exposed to a white noise (60 dB). A mirror was positioned 45° under the ladder in order to videotape (Sony numeric camcorder; HDR-HC3E model) lateral and ventral views of the paws. Distances between rungs were arranged in irregular patterns. To exclude a possible learning effect, the arrangement of the rungs was changed between each session (spacing distance: 2–3 cm). All the animals were exposed to the same pattern within a given session. The animals were tested five times per experimental session. A quantitative evaluation of the faults (total misses, foot-slips and misplacements of the forelimbs) was performed using slow-motion videotape playback. The percentage of foot-faults was initially calculated as follows: (number of footfaults of the affected forelimb X 100)/number of rungs. Data were then expressed as a foot-fault score: (percentage of foot-faults of the affected forelimb)/(percentage of foot-faults of the affected forelimb + percentage of foot-faults of unaffected forelimb). A score greater than 50 was indicative of an impairment of the forelimb affected by the cortical lesion and/or dorsal root section.
- *Forelimb asymmetry test:* the forelimb asymmetry test, or paw preference test, is sensitive to asymmetries produced by different unilateral central nervous system insults [19], [20], [21]. Animals were placed in a clear plastic cylinder (diameter = 20 cm, height = 30 cm) and spontaneous exploratory behavior was videotaped for 3 minutes. The degree of forelimb-use asymmetry was determined by quantifying the use of the left or right forelimb during vertical movement leading to placing the paws on the cylinder wall. During a rear, the first limb to contact the wall was scored as an independent placement for that limb. A subsequent placement of the other limb on the wall while maintaining the initial placement was not scored as an independent movement. Then, an asymmetry index was determined: AI = (affected – unaffected forelimb use)/(affected + unaffected forelimb use). An AI lower than 0 indicated a preferential use of the unaffected forelimb for postural support.
- *Adhesive removal test:* the magnitude of asymmetry in somatosensory sensitivity was evaluated by using the bilateral tactile stimulation test [19,22.23]. With animals placed in a cage without bedding, one strip of adhesive tape (5×5 mm) was applied on the plantar surface of each forepaw, and the time to remove each strip was measured (up to a maximum of 5 minutes). Animals were tested five times per session with a 10 minute rest between successive trials. The order of adhesive placement (left or right paw) was alternated between consecutive trials. An asymmetry index (AI) was then calculated using removal times averaged for each paw, such that AI = (average time for affected paw)/(average time for affected paw + average time for unaffected paw)]. An AI greater than 0.5 was indicative of a susbstantial somatosensory deficit of the paw affected by the cortical lesion and/or dorsal roots section.

### Statistical analysis

Statistical analysis was performed using the Statistica 8.0 software package (StatSoft, Tulsa, OK). Multiple analysis of variance (MANOVA) was employed to evaluate the effects of experimental group and postoperative time. MANOVA was supplemented with multiple comparisons (Newman-Keuls *post-hoc* test). A *P* value less than 0.05 was considered statistically significant. Results are presented as mean ± standard deviation.

## Results

Histological data obtained at each survival time (3, 11 and 30 days) were used to compare tissue loss and gliosis volumes between the I-SE, I-EE and IR rats. As the I-EE rats were housed in EE 24 hours after the cortical lesion, they were not included in the histological analysis performed on the third postoperative day. In addition, since the R and S rats had not been subjected to a cortical lesion, only a qualitative assessment of the cortical tissue was performed for these groups.

### Lesion and gliosis volumes

We first examined the questions of whether the tissue loss and gliosis volumes would evolve between the 3rd and the 30th day, and whether the sensorimotor experience would impact on these parameters.

#### Tissue loss volume

**Figure 2:**
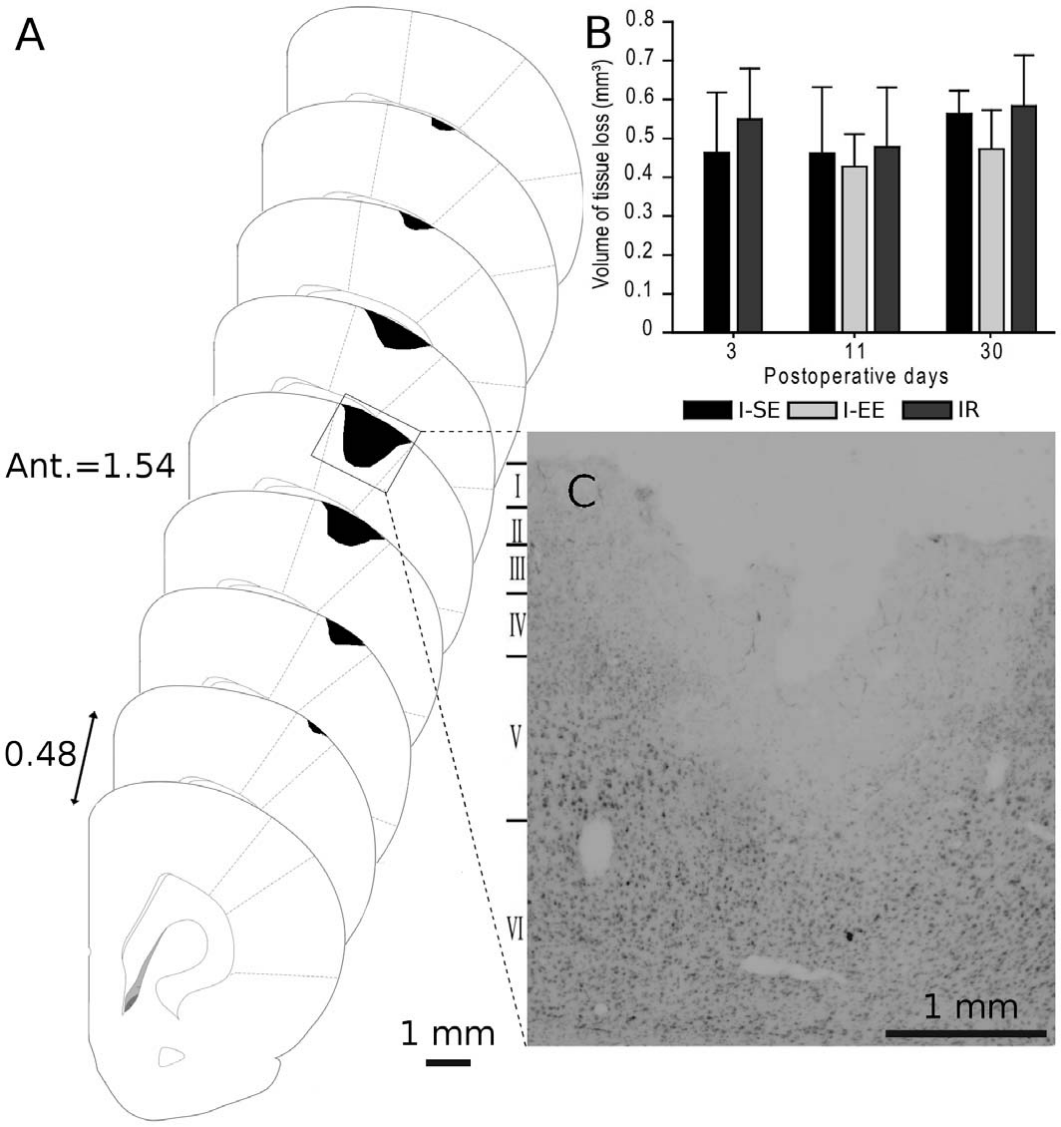
Cortical lesion. (A) Schema of coronal sections reconstructed at 0.48 mm intervals illustrating the rostro-caudal extension of the cortical lesion and adapted from [57]. The region of the cortex destroyed by the cortical compression is illustrated by the shaded areas. (B) Mean values (± standard deviation) of the cortical tissue loss volumes assessed in rats housed in standard (I-SE), enriched environment (I-EE) or subjected to C5–C6 dorsal rhizotomies (IR) at the 3^rd^, 11^th^ and 30^th^ day after cortical compression. (C) Representative coronal brain section stained with cresyl violet showing the lesion of the sensorimotor cortex forelimb area. Scale bar = 1 mm.

All animals subjected to cortical injury showed a lesion restricted to the S1-FL cortex (Fig. 2A–B) that encompassed cortical layers I to IV, while sparing lower layers and the corpus callosum (Fig. 2C). In the S and R rats, no cortical tissue damage was detected. ANOVA showed no main effect of group (F(2, 36) = 1.64, p = 0.21) or postlesion time (F(2, 36) = 1.87, p = 0.17), nor an interaction between these factors (F(4, 36) = 0.30, p = 0.87) thus indicating that the volume of tissue loss remained stable over time in all groups, regardless of the postlesion experience (Fig. 2B).

#### Gliosis volume

**Figure 3:**
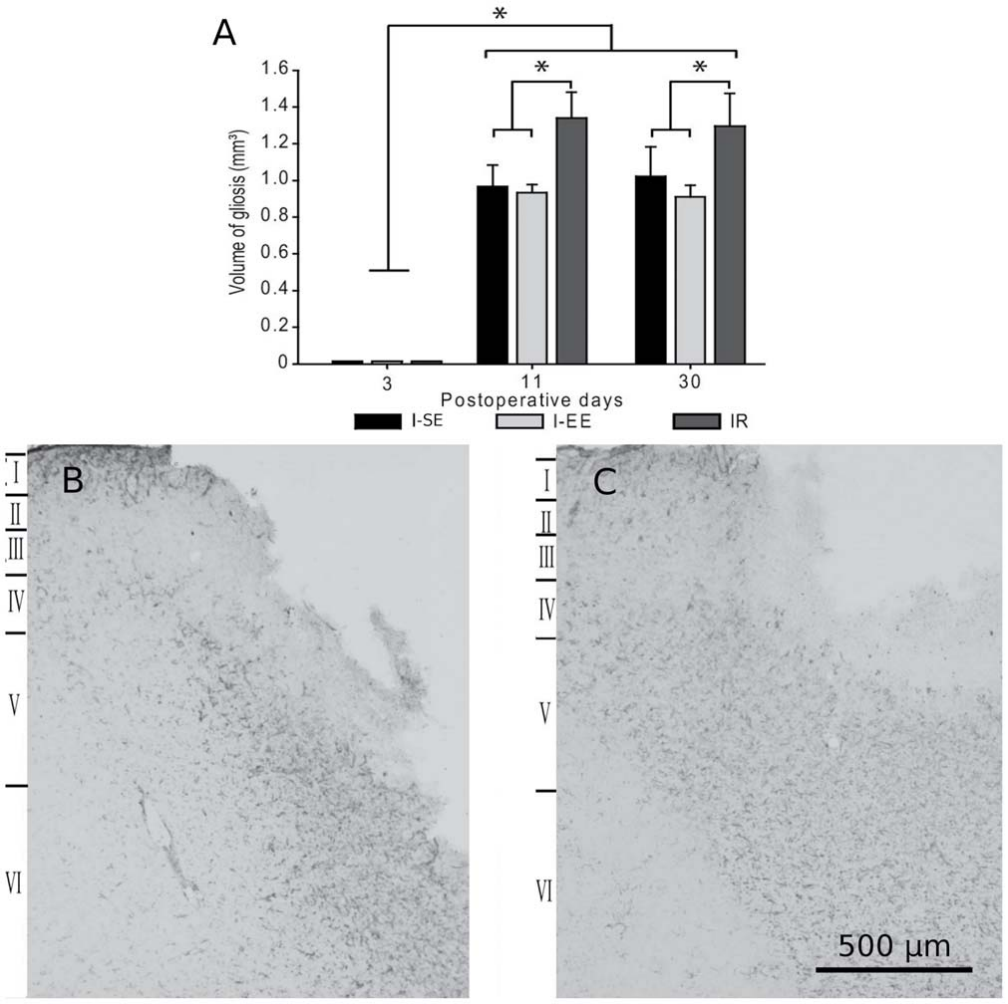
Cortical gliosis. (A) Mean values (± standard deviation) of gliosis volumes assessed at the 3^rd^, 11^th^ and 30^th^ day after cortical compression in rats housed in standard (I-SE), enriched environment (I-EE) or subjected to C5–C6 dorsal rhizotomies (IR). Microphotographs of GFAP-Ir surrounding the lesion area in the I-SE (B) and IR (C) rats, 30 days after the cortical compression. Scale bar = 500 microns.

GFAP is the cell-specific intermediate filament in astrocytes and up-regulation of its synthesis is considered as a reliable indicator of activated astroglia. ANOVA yielded a main effect of group (F(2, 36) = 27.31, p<0.0001) and postlesion time (F(2, 36) = 534.27, p<0.0001) as well as an interaction between these factors (F(4, 36) = 7.14, p<0.0005). A prominent gliosis in the area surrounding the acellular zone was recorded from the 11th day post injury in all lesioned groups (p<0.001) while no gliosis was found on the 3rd day (Fig. 3A).The gliosis volume did not evolve between the 11th and 30th postlesion day in any of the groups (Fig. 3A). Housing in EE did not affect the gliosis volume after cortical injury, whereas the dorsal root section combined with the cortical lesion led to greater gliosis volumes (Fig. 3A–C). By contrast, the rhizotomy itself did not affect the cortical expression of GFAP. In the S rats, the area of cortex exposed during the craniotomy displayed limited GFAP-Ir along the pia mater.

### Behavioral assessments

We examined whether the surgical procedure (parietal craniotomy) or the EE would affect the behavioral performances in rats that were not submitted to cortical lesion, i.e. in the control (C), sham (S; parietal craniotomy), and sham enriched (S-EE) groups. In all the behavioral tests, ANOVA used to compare these groups yielded no main effect of group, postlesion time, nor an interaction between these factors. Therefore, the S group was taken as the reference for further statistical comparisons and illustrations. Then, we determined whether sensory enrichment versus deprivation would result in significantly different time courses of sensorimotor recovery following S1-FL injury. Because of the inability of the R and IR groups to be tested on the second postoperative day, an ANOVA aimed at comparing the initial deficits of the I-SE, I-EE and S groups was used. ANOVA yielded a significant main effect of group in the beam walking (F(2,12) = 109.22, p<0.0001), paw preference (F(2,12) = 715.81, p<0.0001) and adhesive removal tests (F(2, 12) = 266.74, p<0.0001). Post-hoc comparisons indicated, for all three tests, that the I-SE and I-EE groups, which did not differ from each other, exhibited severe deficits on the second postoperative day in comparison to the S rats. Then, we compared the time course of recovery of the I-SE, I-EE, IR, R and S groups from the 5th to the 30th days postlesion. As the performances of IR and R groups were found to be comparable in all tests over the entire period, the IR group is the only one shown in the charts.

#### Foot-faults on the horizontal ladder beam walking test

**Figure 4:**
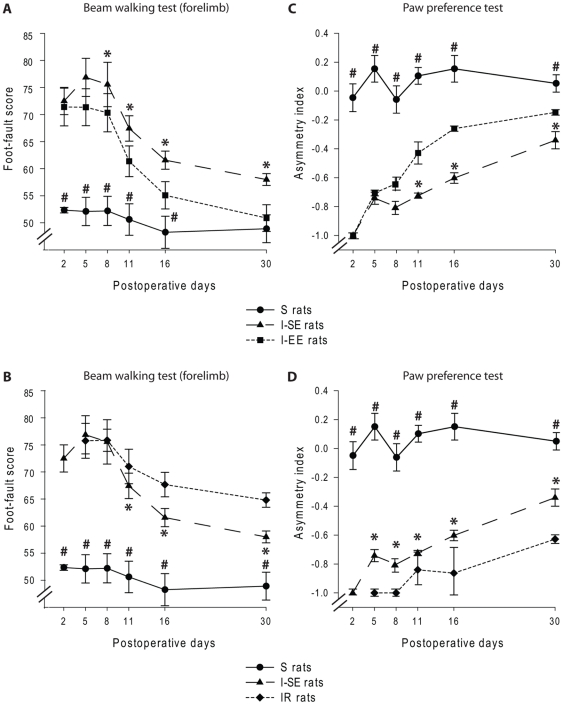
Effect of sensory experience on sensorimotor recovery. Postoperative time course of changes in sensorimotor abilities, assessed using ladder beam walking (A, B) and paw preference tests (C, D). Data are illustrated for injured rats housed in standard (I-SE) or enriched environment (I-EE) relative to Sham (S) rats (A, C) as well as for S and I-SE rats relative to injured rats subjected to C5–C6 dorsal rhizotomies (IR) (B, D). (A) The I-SE group exhibited similar foot-fault score in the beam walking as the I-EE until the 8^th^ postoperative day. After this period, the I-EE performances were significantly better than those of the I-SE group (p<0.05) and reached those of the S group 30 days after injury. (B) The I-SE group exhibited similar foot-fault score as the IR groups until the 11^th^ postoperative days. After this period, the IR performances were significantly lower than those of the I-SE group (p<0.05). (C) During the first postoperative week, the I-SE group exhibited comparable forelimb postural-support that the I-EE. After this period, the I-EE performances were significantly better than those of the I-SE group (p<0.05) but never reached those of the S group. (D) By comparison to the I-SE group, the IR group exhibited significant asymmetrical use of their forelimbs over the entire testing period (p<0.05). Statistical differences between the S and the other groups are shown by #, while differences between the I-SE and I-EE rats (A, C) or I-SE and IR rats (B, D) are indicated by *.

ANOVA used to compare the forelimb performances during beam crossing yielded a significant main effect of group [F(4,100) = 287.59, p<0.0001], postoperative time [F(4,100) = 133.77, p<0.0001] and an interaction between these factors [F(16,100) = 7.39, p<0.0001]. Firstly, the cortical compression was shown to immediately impair the placement of the affected forepaw on the ladder beam, as indicated by a high foot-fault score that resulted in an asymmetrical use of the two forelimbs during stepping in the I-SE group (Fig. 4A). Over time, these rats exhibited a substantial but incomplete recovery, as indicated by a gradually decreasing number of foot-faults for the affected forelimb and a partial recovery of symmetrical use of the two forelimbs during stepping (day 5: 77.03±3.53; day 30: 57.83±1.10; p<0.005) (Fig. 4A).

Secondly, the somatosensory experience appeared to strongly affect the time course of recovery in the beam walking test after the cortical damage (Fig. 4A, B). Whereas no difference between the I-SE, I-EE, R and IR groups were observed during the first postoperative week, these groups exhibited significantly different recovery profiles after this period. Indeed, while the performances of all lesion groups improved with time, as indicated by a gradual decrease in foot-fault scores, the performances of the I-EE group were significantly better than those of the other groups as early as the 8th postoperative day (Fig. 4A). At the end of the postoperative period examined, the foot-fault score observed in the I-EE group (51.08±2.57) was even comparable to that of the S group (48.96±2.57, p = 0.39, n.s.) (Fig. 4A). By contrast, differences between the IR (or R) and the I-SE groups were noted from the 11th (IR: 71.48±3.18, R: 72.72±1.71, *vs.* I-SE: 67.51±2.35) to the 30th postoperative day (IR: 64.40±1.34, R: 63.39±0.84, *vs.* I-SE: 57.83±1.10) as indicated by a slower recovery for the IR (and R) rats (Fig. 4B).

Interestingly, observation of the animals' behavior during the testing sessions suggested that the IR and R rats progressively re-used their affected forelimb but in a different manner in comparison to the I-EE and I-SE rats. After the dorsal root section, the digits were initially retracted into the palm and the animals progressively used their affected forelimb as a “crutch”, a movement strategy that was only observed in these animals.

#### Paw preference test

As also observed for the ladder beam walking test, ANOVA on paw preference data yielded a significant effect of group [F(4,100) = 596.32, p<0.0001], postoperative time [F(4,100) = 89.51, p<0.0001] and interaction between these two factors [F(16,100) = 9.40, p<0.0001]. Firstly, a gradual recovery in the use of the impaired forelimb was observed in the I-SE rats between the 11th and the 30th days (−0.73±0.02 and −0.31±0.06, respectively; p<0.05) (Fig. 4C). However, persistent asymmetries in forelimb postural-support were recorded, as the I-SE group scores significantly differed from those of the S group at the end of the postoperative period examined (S: 0.04±0.06, p<0.0001) (Fig. 4C).

Secondly, the AI were shown to be influenced by sensory experience (Fig. 4C and D). In the I-EE rats, preferential use of the unaffected forepaw for postural support diminished rapidly, from the 11th to the 30th post-lesion day [F(1,8) = 496.19, p<0.001] but asymmetries in forelimb postural-support were not fully compensated for over the postlesion period examined, as indicated by a comparison to S rats (day 30, I-EE: −0.15±0.02; S: 0.04±0.06; p<0.0001) (Fig. 4C). The IR group exhibited a slower, but significant, improvement in the use of their affected forelimb for postural-support compared to the I-SE and I-EE groups, from the end of the first to the fourth postoperative weeks. As illustrated in Fig. 4D, except for the R rats, the performances of the IR and R rats (−0.61±0.03 and 0.54±0.16 on the 30^th^ day, respectively) remained lower than those of the I-SE and I-EE rats during the entire testing period (see also Table S1).

#### Adhesive removal test

**Figure 5:**
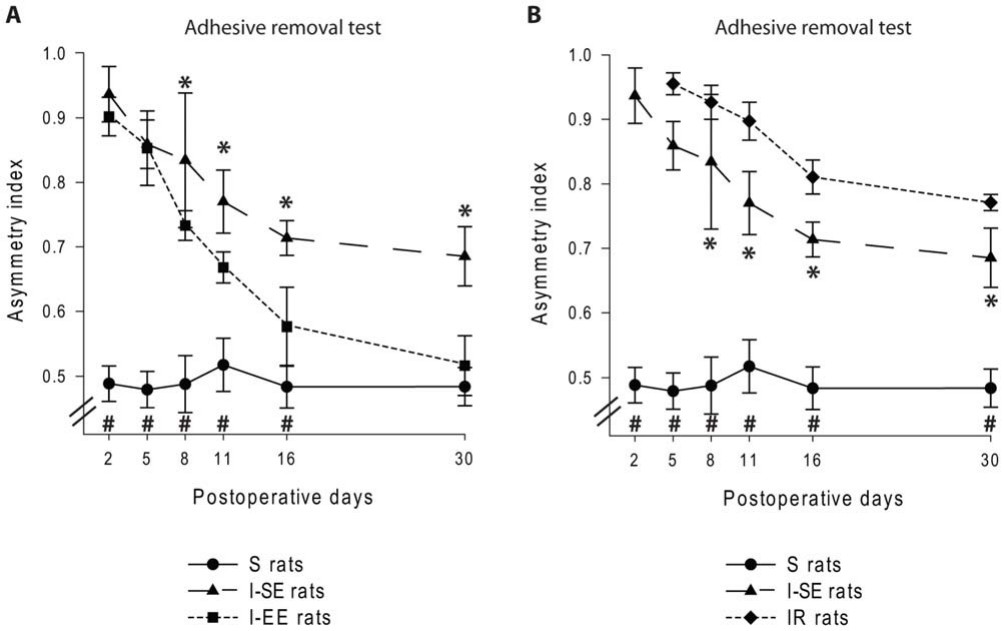
Effect of sensory experience on the recovery of tactile sensitivity. Postoperative time course of tactile abilities assessed using the adhesive removal test in cortical injured rats housed in standard (I-SE) or enriched environment (I-EE) relative to Sham (S) rats (A), as well as in S and I-SE relative to cortical injured rats subjected to C5–C6 dorsal rhizotomies (IR) (B). During the first postoperative week, the I-SE group exhibited similar AI that the I-EE and IR groups. (A) After this period, the I-EE performances were significantly better than those of the I-SE group (p<0.05) and reached those of the S group 30 days after the cortical injury. (B) In contrast, the tactile abilities of the IR group remained significantly lower compared to those of the I-SE group (p<0.05). Statistical comparisons: same conventions as for Fig. 4.

ANOVA indicated significant main effects of group [F(4,100) = 336.19, p<0.0001], postoperative time [F(4,100) = 74.35, p<0.0001] and an interaction between these factors [F(16,100) = 6.52, p<0.0001]. As previously observed with sensorimotor tests, the cortical damage strongly affected the scores obtained in the adhesive removal test. The I-SE rats exhibited tactile deficits that were not fully compensated for during the entire postoperative period, by comparison with the S group (day 30, I-SE: 0.69±0.05; S: 0.48±0.03; p<0.0001). However, and consistent with the other behavioral tests, a gradual recovery was observed between the 8th and the 30th days (day 8: 0.83±0.10; day 30: 0.69±0.05; p<0.0001) (Fig. 5A).

In addition, the degree of tactile deficit recovery was found to be related to the amount of sensory experience (Fig. 5A and B). All the experimental groups, except for the IR and R groups, differed from each other since the 8th postoperative day (day 8, I-EE: 0.73±0.02; I-SE: 0.83±0.1; IR: 0.93±0.03; p<0.05). Compared to the R, IR and I-SE groups, which exhibited comparable recovery profiles (Fig. 5B), but with greater AI for the first two groups (day 5, IR: 0.96±0.02; R: 0.95±0.02; day 30, IR: 0.77±0.01; R: 0.75±0.04) (Fig. 5B), the I-EE group displayed a rapid decrease in sensory asymmetry, such that these rats exhibited similar sensory performance as that of the S group within one month (p = 0.46, n.s.) (Fig. 5A, see also Table S2).

## Discussion

The present study was undertaken to determine whether drastic changes in sensorimotor experience would impact on the postoperative evolution of the lesion and gliosis volumes as well as on the time course and completeness of forelimb sensorimotor recovery after focal S1-FL injury. Our findings show that sensorimotor enrichment, standard housing conditions and severe sensory deprivation resulted in similar lesion sizes over the postoperative period examined. However, sensory deprivation was accompanied by a greater glial reaction. Despite similar tissue loss between the experimental groups, the environmental enrichment, promoting sensorimotor experience, had a beneficial effect on the time course and completeness of forelimb sensorimotor recovery. In contrast, sensory deprivation was found to aggravate the behavioral deficits and to delay the sensorimotor recovery, which remained partial within the postoperative period examined.

### Anatomical effects of sensory experience after unilateral S1-FL injury

Previous reports have shown that extradural cortical compression resulted in an ischemic damage that expanded during the first post-lesion days and remained stable over the subsequent weeks [13], [14]. Such a lesion stability was also observed following our more focal compression. We asked the question of whether drastic differences in sensory experience would influence the lesion size. In previous studies, neither housing in an EE [2], [9], [10] nor sensorimotor restriction of the forelimb impaired by the cortical lesion [5], [12] were shown to affect the infarct size. Consistent with those reports, we found that neither an enriched environment nor a sensory deprivation through dorsal rhizotomies impacted on the tissue damage induced by cortical compression.

In parallel, an increase in GFAP-Ir, indicative of reactive astrogliosis, encompassed the infarct area since the 11th day after the cortical injury. This gliosis volume was found to remain stable afterwards in all the injured groups. These results are in accordance with previous studies that documented similar GFAP-Ir in the peri-infarct area in rats submitted to various types of sensorimotor exercise [4], [8], [9]. An unexpected finding was a greater gliosis volume in the IR rats beginning on the 11th postoperative day, in comparison to I-SE and I-EE rats. This result can be attributed to a higher reactive gliogenesis in the IR rats' cortices. Indeed, recent findings demonstrated the induction of newly generated astrocytes in the primary somatosensory cortex after unilateral cervical dorsal rhizotomy in adult monkeys, suggesting that disruption of activity along the somatosensory pathways is sufficient to induce glial activation within the input-deprived cortex [24]. In this study, the astroglial reaction was too weak to lead to a glial scar. One can hypothesize that the reactive astrogenesis observed in our IR rats, in which dorsal root section and cortical injury were combined, could have enhanced inflammatory processes and thus reinforced subsequent astroglial reaction. Indeed, previous data showed that nerve injury and dorsal root transection resulted in a microglial and astroglial activation within the central nervous system [25], [26].

### Behavioral effects of forelimb sensory experience after unilateral S1-FL injury

In this study, we assessed the behavioral deficits induced by a lesion damaging a discrete and functionally specific cortical area, as well as the consequences of differential intensity of sensory experience, using behavioral tests highly sensitive to motor and sensory impairments. These tests have been used in previous studies analyzing the effects of cortical lesions on behavioral skills [18]–[21].

As described by others, we demonstrated that unilateral cortical compression leads to long-lasting contralateral forelimb misplacements when traversing the beam [21]. The I-SE rats also exhibited impairments of the forelimb contralateral to the cortical lesion and a compensatory increased reliance on the unimpaired forelimb during postural support [21], [27]. As also reported following unilateral cortical aspiration [28], we observed a severe but gradually abating deficit in the ability to respond to tactile stimulation of the forelimb contralateral to the cortical injury during the first postoperative month.

Our findings also indicated that the functional recovery after unilateral S1-FL damage depended on peripheral somatosensory inputs. In the I-SE group, the flow of peripheral afferents generated by the daily activity of the rats in their home cages can be considered as moderate. Deafferentation of the contralateral forelimb by dorsal root section in injured animals resulted in the most severe and long-lasting tactile and sensorimotor deficits. During the entire period examined, the IR and R groups exhibited similar forepaw sensorimotor performances, thus indicating that the delayed recovery observed in these rats, compared to the I-SE rats, can be mainly attributed to the disruption of activity along the forepaw somatosensory pathways. The irreversible deprivation of somatosensory forepaw afferences accounts for the permanent loss of tactile sensitivity, while the moderate recovery of sensorimotor skills underscores the contribution of somatosensory reafferents to fine sensorimotor control [29]–[34].

This severe reduction of sensory experience, imposed on the IR rats, was contrasted with the increased sensory stimulation provided to the I-EE animals. After the focal cortical injury, the early exposure to the enriched sensory environment was shown to improve the recovery of sensorimotor adjustments during beam walking and to facilitate the restoration of symmetrical limb use during rearing behavior. Moreover, the tactile deficits were also fully compensated. Previous studies have documented the beneficial influence of a spontaneous exercise on behavioral recovery after cortical infarct [2], [3], [8], [9], [10], [35]. Moreover, it appears that such a rehabilitative strategy leads to a better functional recovery than that resulting from forced exercise after brain injury. Indeed, an overuse of the impaired forelimb after unilateral cortical injury has been shown to increase the size of the lesion and result in long-lasting deficits in the use of the affected limb [5], [6], [12].

Overall, it appears that the effects of sensorimotor experience on functional recovery after brain insult critically depend on the postlesion time at which this rehabilitative experience is initiated [36]. It has been demonstrated that the beneficial effects of an EE combined with a skilled forelimb reaching training decline with time and improve functional recovery only if initiated 5 days after cortical ischemia [37]. This raises the possibility that a restricted time window exists following cortical insult during which specific rehabilitation strategies have optimal effects, as previously demonstrated following vestibular damage [38], [39].

### Potential mechanisms

Regardless of postlesion environment, similar lesion volumes were reported between rats, indicating that the differential effects of sensory experience on functional recovery after cortical injury cannot be attributed to differences in lesion size. Despite the significant differences observed between the I-SE, IR and I-EE groups in the recovery of sensorimotor performances beginning with the second week following the cortical damage, all of these groups exhibited a gradual improvement over time. As a result, a substantial decrease of asymmetrical forepaw use and tactile sensitivity, as well as a better control of the impaired forelimb during walking was observed. Previous studies have suggested that functional recovery may be attributed to the development of alternate strategies underlying behavioral deficit compensation [5], [40], [41]. After an initial period of neglect, animals in all the injured groups, which were allowed to use both forelimbs, may have developed efficient compensatory strategies over time to elicit proper sensorimotor adjustments while walking on the rungs to cross the ladder, similarly to what we previously described in rats with cervical spinal cord hemisection [42]–[44]. Moreover, one cannot exclude that the modest recovery of both sensorimotor and tactile abilities observed in the R and IR groups could be mediated by a small number of somatosensory fibers spared by the C5–C6 dorsal root section. Indeed, complete deprivation of the whole forelimb afferents would have required more extensive dorsal root sections [45].

Exposing rats to an enriched environment after cortical injury greatly influenced the recovery process, presumably by allowing rats to develop more efficient compensatory behavior as well as by stimulating neuroplasticity mechanisms. Among these mechanisms, growth factors such as NGF, which are over-expressed after EE exposure (for review see [46]), have been shown to play a key role in behavioral recovery following cortical compression [47]. The recovery of the I-EE rats to a prelesion level could be attributed in part to compensatory mechanisms. Nevertheless, the behavioral features observed in these animals that, contrary to the R or IR rats, never exhibited different ways of using their affected forepaw, suggest a restoration of the normal underlying neurophysiological mechanisms. Moreover, it is very likely that the tactile recovery observed in the I-EE rats is not accounted for by behavioral strategies [48], but could be attributed to cortical reorganization within spared somatosensory areas as documented following focal cortical injury [49]–[51]. Indeed, a previous study in monkeys showed that manual skill training induced a partial reactivation or expansion of the contralateral cortical fields representing the skin surfaces of the trained digits in ectopic sectors of area 3b and in area 1, respectively [51]. In addition, new representational sectors for these trained surfaces emerged in cortical territories of area 3a that formerly did not exhibit cutaneous responses. This representational substitution was accompanied by a functional recovery of manual dexterity. Similar results were reported after cortical lesion to the primary motor area [52]. Collectively, these findings indicate that behaviorally relevant stimulations enhance plastic changes within the sensory and motor cortical areas after focal stroke, thereby promoting functional recovery. It has been hypothesized that exposure to EE strengthens neuroplasticity mechanisms at all levels of the central nervous system [53] and induces axonal growth and dendritic sprouting in the vicinity of brain lesions [54]. These newly grown sprouts and their synapses may regain some function [55], thereby mediating the improvement of behavioral performances [56] as revealed by high sensorimotor scores in I-EE animals. In addition, high levels of afferent activity can trigger substitutive mechanisms enabling the recruitment, sometimes temporary, of intact cortical areas spared by the lesion [51].

These findings provide evidence of a beneficial effect of sensory experience as early as the end of the first postlesion week. The functional recovery reported herein may be mediated by the development of behavioral compensatory strategies as well as the functional reorganization of damaged and/or intact cortical areas. Rehabilitative strategies following brain damage should take advantage of an early use of sensory loops to recalibrate and improve sensorimotor adjustments, as well as to stimulate neuroplasticity mechanisms so as to maximize functional recovery.

## Supporting Information

Table S1 Mean scores on paw preference test. The mean number of rearing on a cylinder wall during 3 minute periods is given for each forepaw on the 5^th^, 16^th^ and 30^th^ days after the cortical compression. These values were used to determine the asymmetry index (AI) illustrated in Fig. 4.
(XLS)

Table S2 Mean times on the adhesive removal test. The average of the time (sec) necessary to remove a calibrated strip of adhesive from each forepaw is given on the 5^th^, 16^th^ and 30^th^ days after the cortical compression. These values were used to determine the asymmetry index (AI) illustrated in Fig. 5.
(XLS)
